# Evaluation of a student-led breast cancer awareness campaign as a co-curricular model in pharmacy education: a mixed-methods study in the UAE

**DOI:** 10.1186/s12909-026-09356-8

**Published:** 2026-05-11

**Authors:** Maram O Abbas, Semira Beshir, Doaa Kamal AlKhalidi, Azhar T Rahma, Iffat Elbarazi, Rahaf AL-Zeer, Hana Yahya, Fatima AL-Maskari

**Affiliations:** 1 Institute of Public Health, College of Medicine and Health Sciences, United Arab Emirates University, Al-Ain, United Arab Emirates; 2 College Of Pharmacy, Dubai Medical University, Dubai, United Arab Emirates; 3 Pharmacy College, Gulf Medical University, Ajman, United Arab Emirates

**Keywords:** Experiential learning, Pharmacy education, Student-led health campaigns, Public health promotion, Adolescent health education, Breast cancer awareness, Mixed-methods research, UAE, Experiential learning theory, Kirkpatrick model

## Abstract

**Background:**

Experiential learning in pharmacy education fosters professional competencies through real-world engagement. Despite the high burden of breast cancer in the UAE, adolescents are an under-targeted group in awareness campaigns. Additionally, pharmacy curricula rarely incorporate student-led education initiatives. This study evaluated a student-led breast cancer awareness campaign to explore its educational impact on pharmacy students and short-term knowledge gains among adolescent girls.

**Methods:**

This mixed-methods study, conducted in Dubai, included a qualitative component (two focus groups with 16 final-year pharmacy students) and a quantitative component using a single-group pre–post design with 87 female secondary school students. Thematic analysis guided by the Kirkpatrick Model evaluated training outcomes based on participants’ reactions, learning gains, behavioural changes, and broader results. The quantitative phase assessed knowledge gains among 87 female secondary school students using pre- and post-intervention surveys. Descriptive statistics and the Wilcoxon signed-rank test were used in the data analysis.

**Results:**

Across Kirkpatrick levels, pharmacy students reported strong engagement with the campaign and meaningful experiential learning outcomes, particularly in health communication, advocacy, and professional identity development. Quantitatively, total knowledge scores among school participants increased from 41.7 ± 15.2 to 67.6 ± 15.7 (*p* < 0.001). At the domain level, mean absolute percentage-point gains were + 31.0 for breast-cancer signs, + 23.6 for risk-factor knowledge, and + 34.8 for screening concepts, indicating consistent improvements across domains. Overall, willingness to seek medical help for breast changes increased following the intervention.

**Conclusion:**

The student-led awareness campaign was associated with measurable short-term improvements in breast cancer knowledge among adolescents and meaningful experiential learning outcomes for pharmacy students. Delivered as a co-curricular activity, the initiative illustrates a practical model for integrating student-led health education into pharmacy curricula to enhance advocacy and experiential learning.

**Supplementary Information:**

The online version contains supplementary material available at 10.1186/s12909-026-09356-8.

## Introduction

Experiential learning plays a pivotal role in competency-based pharmacy education, emphasising the integration of knowledge, skills, and professional values through direct real-world engagement [1]. This learner-centred approach aligns with constructivist theory, in which students build understanding through reflection and active participation rather than passive instruction. Frameworks like Kolb’s Experiential Learning Cycle support this process by emphasising concrete experience, reflective observation, conceptual understanding, and practical application [2, 3]. Experiential learning enables pharmacy students to develop key competencies such as interprofessional collaboration, leadership, empathy, and adaptability-traits that are seldom acquired through passive learning [4].

Student-led public health campaigns enable learners to take ownership of health education initiatives while simultaneously addressing community health priorities [5]. These campaigns extend beyond simulated settings by involving students in planning, delivering, and evaluating real interventions to improve population health. Through this process, students move from learners to contributors, shifting their mindset from academic achievement to social accountability [6]. Moreover, such campaigns reinforce the pharmacist’s expanding role as a community health advocate and educator, who must be equipped with pharmacological expertise alongside confidence and competence to lead health promotion efforts [7–9]. What makes this approach pedagogically valuable is its dual function: it is not simply community outreach, but a structured teaching strategy that embeds advocacy, reflection, and leadership training into the pharmacy curriculum. In doing so, it transforms service into a curricular tool for developing professional identity and competencies [10].

In experiential learning design, choosing a real-world issue is crucial to maximising educational impact and public value [11]. Breast cancer (BC) represents an ideal focal point due to its ongoing burden and the preventable nature of many of its consequences [12]. Globally, BC is the most commonly diagnosed cancer among women and a leading cause of cancer-related mortality. In the United Arab Emirates (UAE), BC accounts for nearly 38% of all female cancers and is frequently diagnosed at a younger median age (49 years) and more advanced stages compared to Western populations [13–15]. Despite national screening guidelines recommending biennial mammography for women aged 40–69, screening uptake remains low, with only 14–23% of eligible women having ever undergone mammography [16, 17]. This combination of high disease burden, earlier onset, and low screening participation underscores the urgency of implementing targeted and culturally relevant awareness strategies.

Despite ongoing BC awareness initiatives in the UAE, such as the Pink Caravan ride and government programs, significant knowledge gaps persist among women [18]. These campaigns focus more on adult women eligible for screening and early detection than on adolescents. Cultural stigma, modesty norms, and taboos surrounding breast health further restrict open discussions in the UAE society, leading to embarrassment, misinformation, and delayed help-seeking behaviours among young females [17, 19–22].

Targeting adolescents, particularly school-aged girls, is therefore essential for fostering preventive health attitudes and building foundational knowledge that can inform future decision-making [23, 24]. Educating this demographic early also enables intergenerational transfer of awareness within families, as younger generations often influence health beliefs and practices at home [25, 26].

While student-led health campaigns have improved learner competencies and community awareness in countries such as Malaysia, Nigeria, and the United States [4, 27–29], similar initiatives remain scarce in the UAE. For example, U.S. pharmacy programs embed public health outreach activities into co-curricular requirements to meet Accreditation Council for Pharmacy Education (ACPE) standards, strengthening students’ communication and health promotion skills through direct community engagement [30].

Emerging evidence from low- and middle-income settings comparable to the GCC/MENA region suggests that school-based or peer-led interventions can effectively foster early help-seeking intentions and behaviours among adolescents, supporting the integration of such programs into school curricula [31, 32]. Furthermore, this gap features the relevance of our mixed-methods study in Dubai.

In the UAE, pharmacy education has recently shifted towards competency-based frameworks aligned with national standards, with a focus on patient care, professionalism, and communication skills [33]. However, UAE pharmacy curricula still face constraints on integrating student-led initiatives, including a lack of curricular flexibility, cultural sensitivities regarding breast health, and an emphasis on biomedical sciences over structured community advocacy training [34–36]. The scarcity of student-led advocacy initiatives targeting adolescents represents a missed opportunity to train pharmacy students to be effective health educators who can foster culturally sensitive awareness and prevention.

Accordingly, this study aimed to evaluate a pharmacy student-led BC awareness campaign by exploring its dual impact: first, on the experiential learning outcomes of pharmacy students using the Kirkpatrick Model; and second, on the BC knowledge and attitudes of female secondary school students in Dubai. By examining both educational and public health dimensions, this research suggests that student-led interventions can simultaneously enhance professional learning and community well-being, aligning with pharmacy education reform and health promotion priorities in the region. The findings may inform pharmacy educators seeking to integrate experiential learning and advocacy training into curricula, as well as health policymakers designing culturally relevant BC awareness strategies targeting adolescents.

## Methods

### Study design & setting

This study employed a convergent parallel mixed-methods design, in which qualitative and quantitative data were collected and analysed concurrently to provide a comprehensive understanding of both pharmacy students’ learning outcomes and secondary-school students’ knowledge gains. This design is widely used in pharmacy and health professions education [37] enabled the integration of measurable outcomes with deeper experiential insights. By analysing both data types in parallel and merging them at the interpretation stage, the study addressed two complementary questions: (1) how the campaign influenced adolescents’ breast cancer knowledge and attitudes, and (2) how pharmacy students experienced the initiative as a learning activity. This approach ensured that qualitative reflections contextualised quantitative results, and that both perspectives contributed equally to understanding the campaign’s overall impact.

The quantitative component used a pre- and post-intervention design to measure changes in knowledge and attitudes among female secondary school students. The qualitative component explored the experiential learning outcomes among final-year pharmacy students who implemented the intervention.

A convenience sampling method was employed to recruit secondary schools based on willingness and accessibility. Although convenience sampling facilitated recruitment and timely data collection, it inherently limits the generalisability of the findings. All four participating institutions were private secondary schools, comprising two Arabic-language and two English-language curricula, representing the main educational models within Dubai’s private school system. While the absence of public schools restricts generalisability, the inclusion of both language streams reflects the bilingual educational context typical of the UAE. Public schools were not included due to administrative and governance approval requirements, which could not be accommodated within the study timeframe.

The campaign was embedded within the schools’ extracurricular health education activities and implemented collaboratively by academic researchers and final-year pharmacy students as part of a structured experiential learning module. The intervention was conducted in October and November 2023.

Ethical approval for this study was obtained from the Research and Ethics Committee (REC/FR/2023-24/06) of Dubai Pharmacy College for Girls. Informed consent was obtained from all participating students and guardians.

### Study participants

#### Pharmacy students

The qualitative phase included 16 final-year pharmacy students from the pharmacy college enrolled in a community outreach unit. Inclusion criteria were: (1) active participation in the BC awareness workshop delivery, and (2) willingness to engage in post-campaign focus group discussions. Students were briefed about the research aims and provided written informed consent.

The sample size of 16 students was determined based on guidance for focus group research aimed at capturing diverse perspectives within homogeneous professional student groups. Two focus groups (*n* = 8 each) were conducted, aligning with methodological recommendations that suggest two to three focus groups are often sufficient to achieve thematic saturation [38]. Saturation was assessed iteratively, with transcripts reviewed after each focus group. In the second group, no new themes emerged, and existing categories were well developed, suggesting that thematic saturation had been reached [39].

#### Secondary school students

A total of 87 female students aged 16–18 years were recruited through convenience sampling. Inclusion criteria were: (1) enrollment in Grade 11 or 12, and (2) provision of parental consent and student assent. Within each selected school, all students who provided parental consent and were present on the campaign day were included, and no additional selection occurred at the student level.

Although a formal power analysis was not conducted due to the study’s pilot and educational nature, the sample size (*n* = 87) included all eligible students available at the participating schools during the campaign period. Similar school-based health education interventions have employed comparable sample sizes to detect significant changes in knowledge [24]. Pilot-study guidelines recommend 12–50 participants per group to estimate variance and refine procedures [40], while CONSORT-Pilot advises that feasibility objectives, rather than hypothesis testing, should dictate the number of participants [41]. Our sample, therefore, exceeds these minimums.

### Intervention

Prior to outreach, pharmacy and public health faculty briefed students on BC epidemiology, risk factors, screening guidelines, and early detection; a pharmacy education specialist then trained them in culturally sensitive, adolescent-focused health communication techniques.

The campaign was implemented as a structured workshop featuring:

- *Educational Presentation*: A comprehensive slide presentation, reviewed by faculty and an oncologist and based on CDC and WHO resources, covered BC symptoms, risk factors, screening methods, and early detection [42, 43].
- *Interactive Question-and-Answer Session*: An interactive segment encouraged dialogue and clarified misconceptions, reinforcing the workshop’s key messages.
- *Interactive Learning Activities*: “Myth or Fact” competitions and small group quizzes promoted critical thinking and deeper understanding.
- *Group Discussion and Reflection*: Participants shared insights and reflections, fostering engagement and reinforcing key awareness messages.

To ensure consistency and standardisation across schools, all student facilitators used the same pre-approved slide deck, facilitation guide, and structured agenda. Faculty supervision during sessions further ensured consistent delivery and content accuracy.

To maintain cultural appropriateness, the campaign was delivered exclusively by female pharmacy students in female-only settings within the schools. Language and educational materials were carefully adapted to respect local modesty norms and cultural beliefs about breast health. Visual content featured stylised silhouettes rather than anatomical photography to avoid discomfort while ensuring clarity, and educational descriptions employed culturally acceptable terminology to facilitate respectful and effective communication. These adaptations aimed to enhance students’ confidence in discussing breast health topics that are often considered taboo and to promote culturally competent health education.

### Data collection

#### Qualitative phase

Within two weeks of the campaign’s conclusion, two semi-structured online focus groups were convened, each comprising eight pharmacy students. Sessions were conducted via Microsoft Teams and lasted 60–90 min. All discussions were conducted in English, audio-recorded with informed consent, transcribed verbatim, and subsequently anonymised.

The detailed semi-structured interview guide was explicitly developed and mapped to the four levels of the Kirkpatrick Model (Table S1, Supplementary) [44]. To evaluate students’ experiential learning systematically. Topics addressed included campaign engagement, knowledge acquisition, behavioural changes, and perceived impacts on both participants and the target community.

Given the potential influence of power dynamics in student–faculty relationships, the focus groups were conducted and analysed by an independent qualitative researcher with no teaching or assessment role in the college. This approach was intended to minimise social desirability bias and encourage open reflection [45].

Data were analysed thematically, following Braun and Clarke’s six-phase approach, within a deductive framework informed by the Kirkpatrick Model. The independent researcher maintained analytic memos and a reflexive journal to document positionality, analytic decisions, and evolving interpretations throughout the process. Reflexivity was further supported through the researcher’s use of field notes to capture assumptions, methodological reflections, and contextual nuances, consistent with best practice in qualitative inquiry [46].

The qualitative component was designed and reported per the Consolidated Criteria for Reporting Qualitative Research (COREQ) 32-item checklist, thereby ensuring methodological transparency, analytical rigour, and reproducibility (Table S4, Supplementary) [47].

#### Quantitative phase

Data were collected from secondary school students using a validated, self-administered questionnaire administered immediately before and directly after the workshop on the same day to minimise external influences. The surveys were distributed and collected by pharmacy students involved in the campaign under the direct supervision of faculty members. Each participant received both pre- and post-workshop questionnaires, which were assigned a unique alphanumeric identifier to enable response pairing while preserving anonymity. No identifying personal information was recorded, and completed questionnaires were handled confidentially. Survey completion was supervised in the classroom to ensure clarity of instructions and prevent discussion among participants.

All questionnaire items were mandatory, and no missing demographic data were recorded. Survey items were adapted from previously validated regional breast cancer knowledge and awareness instruments [20, 43, 48]. To ensure both cultural and contextual relevance to the UAE setting. The final instrument comprised three domains assessing: (1) knowledge of breast cancer signs and symptoms (8 items), (2) knowledge of risk factors (16 items, including modifiable, unmodifiable risks, and potential risks), and (3) knowledge of screening and early detection practices (8 items). Each correct response was scored as 1, and incorrect or “do not know” answers were scored as 0;. Domain scores were summed to generate a total knowledge score (maximum = 32).

The original survey was developed in English and then translated into Arabic by a bilingual public health researcher, followed by a back-translation into English by a separate academic to ensure linguistic and conceptual equivalence. Internal consistency reliability in the present sample was satisfactory (Cronbach’s α = 0.78 for signs, 0.81 for risk factors, 0.76 for screening, and 0.81 for the total scale). The instrument’s domain structure, item content, correct-response key, and scoring rules for multi-response questions are provided in Supplementary Table S2 and Table S3.

### Theoretical framework

The study design was guided by experiential learning theory, which emphasises that learners build knowledge most effectively through active participation, reflection, and application in authentic contexts. This theory was relevant to the study, as it informed the campaign’s structure, ensuring that pharmacy students were not passive recipients of information but were actively involved in planning, delivering, and evaluating a real-world health education initiative.

In parallel, the Kirkpatrick Model served as the evaluation framework for systematically assessing the outcomes of the experiential learning intervention [49]. Developed initially to assess training effectiveness in organisational settings, it has been extensively adapted to evaluate learning interventions in academic and healthcare environments [50–52]. It offers a structured, four-level approach:

1. Reaction, measuring participants’ engagement and satisfaction
2. Learning, assessing knowledge, skills, or attitudes acquired.
3. Behaviour, evaluating the transfer of learning to practice.
4. Results, examining broader outcomes or impacts resulting from the intervention.

While Level 4 traditionally measures organisational or system-level outcomes, in educational and healthcare research, it is often interpreted as learners’ perceived broader impact, for example, community influence, professional identity development, and the transfer of learning into future practice. This adaptation is particularly appropriate in educational contexts where institutional or population-level outcomes cannot be directly measured.

Although the model effectively captures participant satisfaction and short-term learning gains (Levels 1–2) in public-health education, evidence of sustained behavioural or population-level change (Levels 3–4) remains limited due to methodological and contextual challenges. Thus, the model serves well as an early indicator of learning impact but requires complementary longitudinal evaluation to confirm sustained public-health effects [53, 54].

### Data analysis

#### Qualitative analysis

Transcripts were analysed using thematic analysis; the Kirkpatrick Model was selected as the guiding framework for evaluating pharmacy students’ experiential learning outcomes due to its widespread application and adaptability in health professions education [55].

A deductive coding approach was used, with an a priori codebook structured around the four Kirkpatrick Model levels — Reaction, Learning, Behaviour, and Result — to systematically categorise the data. These four levels formed the main themes, within which subthemes were identified to capture specific dimensions of students’ experiences. Coding was conducted using NVivo 12 by two researchers independently, with discrepancies resolved through consensus discussions. Cohen’s kappa = 0.79 indicates inter-coder reliability. Themes were illustrated with anonymised quotes and visualised through an AI-generated concept to enhance presentation clarity without influencing coding or analysis.

#### Quantitative analysis

Data was analysed using IBM SPSS version 29. Descriptive statistics (frequencies, percentages, means, SDs) were used for demographic variables. Pre–post differences in knowledge scores were analysed using the Wilcoxon signed-rank test, due to the non-normal distribution of post-test scores (verified by Shapiro-Wilk and Kolmogorov-Smirnov tests). Accordingly, the main text emphasises domain-level changes (signs, risk factors, screening) using absolute percentage-point differences and 95% confidence intervals where appropriate, while the full item-level McNemar results are presented for completeness. This approach mitigates inflation of Type I error without implying confirmatory inference.

Knowledge categories (≤ 50, 51–80, > 80) were used for descriptive display; continuous outcomes remained the primary focus of the analysis. These percentage-based thresholds reflect a standard convention in KAP and health-education research and serve only as interpretive aids rather than psychometric cut-offs [50].

## Results

### Qualitative phase

**Fig. 1:**
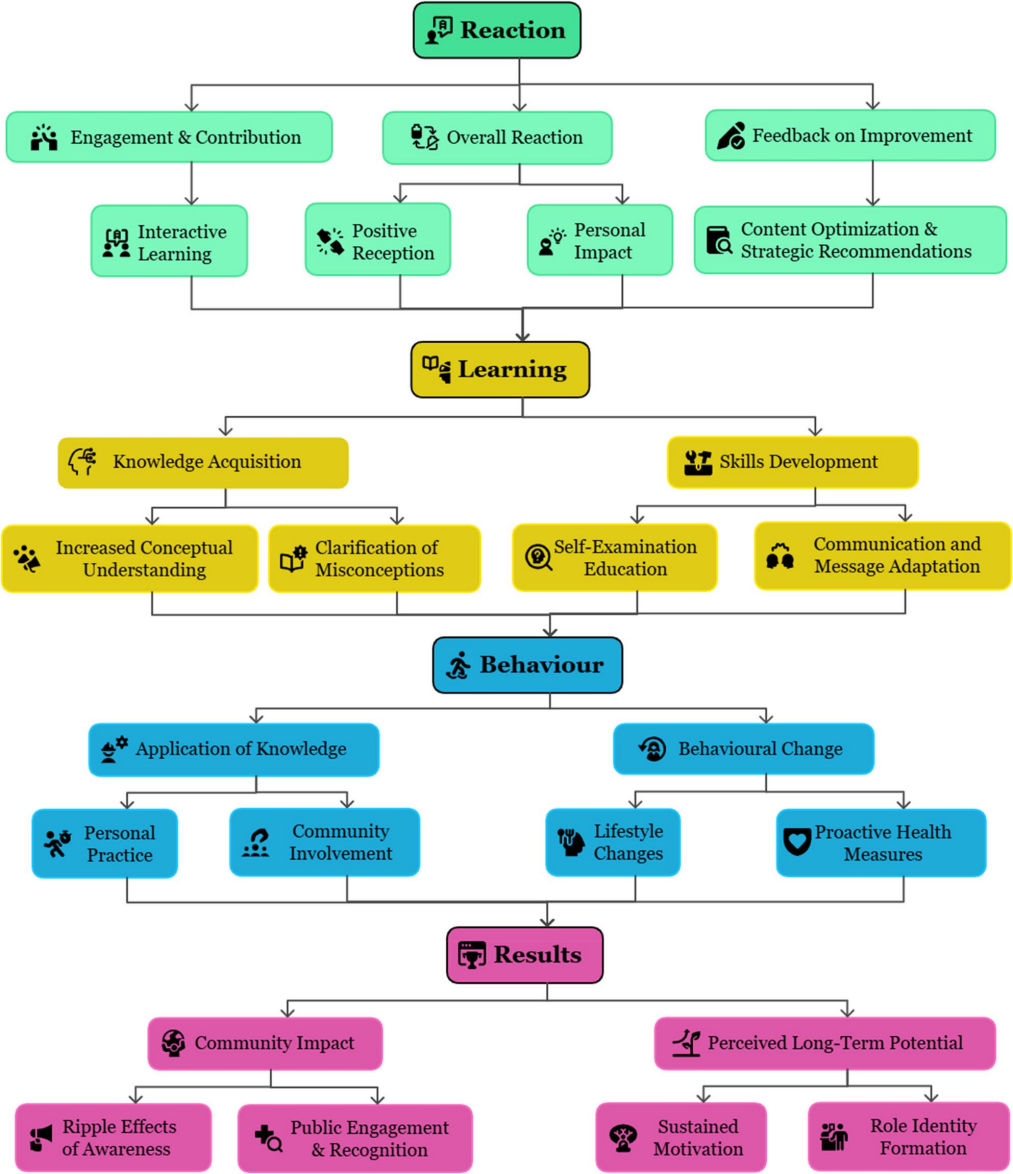
Mapping Learning Outcomes of a Breast Cancer Awareness Campaign: Kirkpatrick Framework

The Kirkpatrick Model was used to evaluate the educational impact of a student-led intervention, providing a structured framework for exploring students’ experiences across four levels: reaction, learning, behaviour, and results. Widely used in medical education, the model aligns well with competency-based training by assessing knowledge gain, behavioural transformation, and societal contributions [44, 49, 56]. Figure 1 provides a conceptual mapping of the themes and subthemes generated from our analysis, illustrating how students’ reflections aligned with the four levels of the Kirkpatrick framework. Table 1 summarises representative participant quotations organised by Kirkpatrick level, theme, and subtheme to illustrate key experiential insights.

**Table 1:** Illustrative quotations supporting key themes identified across the four Kirkpatrick levels

Kirkpatrick Level	Theme	Subtheme		Representative Quotation (participant ID)
Level 1 – Reaction	Engagement and Contribution	Interactive engagement and active facilitation		“It enhanced my understanding of various diseases and was personally meaningful to me.” (P4, FG2) “During the workshop, the students appeared to reconsider their views, recognising that some of their prior beliefs were based on media portrayals rather than factual information.” (P7, FG1) “Initially, capturing the audience’s attention was challenging; however, the incorporation of interactive games and small incentives increased their engagement.” (P2, FG2)
	Overall Reaction	Positive reception		“It is essential to sustain breast cancer awareness initiatives and facilitate ongoing dialogue throughout the year.” (P7, FG2)
		Personal impact and role identity		“Prior to volunteering in this campaign, I had not fully recognised the importance of breast cancer awareness; this experience underscored its significance for me.” (P1, FG1)
	Feedback on Improvement	Content optimisation and strategic recommendations		“Future awareness initiatives should adopt an inclusive approach by engaging diverse community groups, such as men and parents… to dispel misconceptions, encourage early detection, and ensure that awareness messages are relevant to all.” (P8, FG2)
Level 2 – Learning	Knowledge Acquisition	Increased conceptual understanding		“I sought additional information to deepen my understanding of the disease and its underlying mechanisms.” (P4, FG1)
				“It was a wonderful experience full of new information.” (P2, FG1)
				“Delivering health education to others became a simultaneous learning opportunity for us, reinforcing our own understanding through teaching.” (P3, FG2)
		Clarification of misconceptions		“We corrected many misconceptions we did not realise we had before this experience.” (P4, FG2)
	Skills Development	Self-awareness education		“I helped my colleagues understand the importance of breast self-awareness and how to communicate it effectively.” (P5, FG1)
				“The campaign equipped me with the confidence and skills to educate my family and friends about breast self-awareness.” (P1, FG2)
		Communication and message adaptation		“We usually used medical terms, but we realised we had to simplify them so they could understand.” (P4, FG1)
Level 3 – Behaviour	Application of Knowledge	Personal practice		“I now possess greater knowledge of breast self-awareness and have actively shared this information with my family and friends.” (P8, FG1)
		Community involvement		“We organised additional awareness sessions in our community to keep the conversation going beyond the campaign.” (P5, FG2)
	Behavioural Change		Lifestyle changes	“I adopted lifestyle modifications, including increased physical activity and a healthier diet, to reduce my personal risk profile.” (P6, FG1)
			Proactive health measures	“I have become more aware of my health and regularly check for any signs of changes.” (P1, FG2)
Level 4 – Results	Community Impact	Ripple effects of awareness		“One student said, ‘I want my mum to do the screening now,’ and it made me realise how our words influence others.” (P6, FG1)
				“Several participants sought further information by engaging with our campaign’s social-media platforms, indicating sustained interest.” (P3, FG2)
		Public engagement and recognition		“We felt like professionals; they asked questions and waited for our answers as if we were reliable healthcare providers.” (P2, FG2)
	Perceived Long-Term Potential	Sustained motivation		“Even after the event, I thought of ideas to improve it next year. I want to reach more people.” (P6, FG2)
				“We should do this regularly, not just once. Awareness should not stop.” (P7, FG1)
		Role-identity formation		“Previously uncertain about my career path, I now recognise the meaningful impact pharmacists can have in public health.” (P8, FG2)
Challenges and Unexpected Findings				“It took a while to get them to respond, but once they started participating, the discussion flowed much better.” (P6, FG1)

#### Level one: reaction

At this level, students reported high engagement and found the campaign relevant to their future roles. Moreover, students demonstrated strong emotional and cognitive engagement with the campaign, highlighting its authenticity and impact. The hands-on nature, through role-play, interactive activities, and real-world health communication, shifted learners from passive recipients to active contributors. Their responses reflected enthusiasm, a sense of purpose, and a growing professional identity.

#### Themes and subthemes

- ❖ Engagement and Contribution
  - Interactive Engagement and Active Facilitation: Students highlighted the campaign’s dynamic structure, particularly its use of gamified elements such as puzzles and Q&A sessions, as central to maintaining attention and enhancing message retention. These interactive components made learning enjoyable for school participants and empowered the pharmacy students to step confidently into educator roles. By actively delivering content and facilitating discussions, students developed a sense of ownership, strengthened their communication skills, and gained real-world experience in health promotion.
- ❖ Overall Reaction
  - Positive Reception: The campaign was widely appreciated for its relevance and personal resonance, particularly given the sensitive nature of the topic.
  - Personal Impact and Role Identity: Several students described a transformative shift in their understanding of public health roles, highlighting how participation deepened their sense of responsibility and shaped their future career goals.
- ❖ Feedback on Improvement
  - Content Optimisation and Strategic Recommendations: Participants recommended refining the content and delivery to better resonate with varied audiences, including underrepresented groups such as mothers and men. They also stressed the importance of increasing the frequency and reach of such initiatives.

#### Level two: learning

Key findings at this level included enhanced conceptual understanding of BC and the development of critical health communication skills. This level assesses the knowledge and skills that pharmacy students gain through their engagement in the campaign. The intervention’s experiential design created authentic learning opportunities, enabling students to apply theoretical knowledge in a practical, community-based context —an essential aim of competency-based pharmacy education.

#### Themes and subthemes

- ❖ Knowledge Acquisition
  - Increased Conceptual Understanding: Students stated a strengthened grasp of breast cancer-related topics, including early detection methods, risk factors, and preventive strategies. The campaign enabled them to translate academic content into accessible public health messages.
  - Clarification of Misconceptions: Facilitating the campaign prompted students to confront and correct common myths about BC, reinforcing their learning while educating others.
- ❖ Skills Development
  - Self-Awareness Education: Participating students testified to increased confidence in explaining and demonstrating breast self-awareness techniques, both verbally and visually, which reinforced their comprehension.
  - Communication and Message Adaptation: Delivering complex health information to younger, non-medical audiences enhanced students’ ability to simplify technical terms, navigate sensitive topics, and engage meaningfully, skills critical for future pharmacist roles.

#### Level three: behaviour

Key findings at this level reflected the application of learning to personal practices, health behaviours, and community engagement. This level illustrates how participants integrated new knowledge into daily routines and outreach activities, core indicators of experiential learning in health education.

#### Themes and subthemes

- ❖ Application of Knowledge
  - Personal Practice: Students incorporated breast self-awareness into their routines and encouraged family and peers to do the same.
  - Community Involvement: Several participants took initiative beyond the initial campaign by organising follow-up awareness sessions and disseminating information through informal networks.
- ❖ Behavioural Change
  - Lifestyle Changes: Participation in the campaign prompted healthier habits, such as improved diet choices and increased physical activity.
  - Proactive Health Measures: Students became more attentive to early-detection practices, performing routine self-checks and seeking timely consultation with healthcare professionals when concerned.

#### Level four: results

Key findings at this level reflected participants’ perceptions of broader community influence, evolving professional identity, and motivation for continued advocacy. While Kirkpatrick Level 4 traditionally denotes long-term organisational outcomes, it is commonly adapted in educational research to represent learners’ perceived broader impacts and the transfer of learning into their emerging professional roles. Within this context, the findings indicate early markers of potential broader influence rather than confirmed systemic or long-term change.

#### Themes and subthemes

- ❖ Community Impact
  - Ripple Effects of Awareness: Students reported that their outreach activities sparked interest and discussion about breast health among schoolgirls, families, and peers. Some noted that community members expressed intentions to seek screening or learn more following the sessions.
  - Public Engagement and Recognition: Participants experienced being approached for information and guidance, which reinforced their confidence and sense of credibility as emerging health advocates.
- ❖ Perceived Long-Term Potential
  - Sustained Motivation: Although objective follow-up was beyond the study’s timeframe, many students expressed enthusiasm to continue organising awareness campaigns and apply their communication skills in future practice, suggesting an enduring motivational effect.
  - Role Identity Formation: The campaign experience deepened students’ identification with the pharmacist’s public-health role and, for some, influenced career aspirations toward community or public-health pharmacy.

### Challenges and unexpected findings

During the campaign, pharmacy students reported a range of challenges, including difficulties in effectively engaging adolescents, navigating cultural sensitivities when discussing breast health, simplifying complex medical terminology into age-appropriate language, and managing limited time within school schedules. Despite prior training, some students also identified gaps in their BC knowledge, prompting further self-study.

### Quantitative phase

#### School students demographics

Eighty-seven female secondary school students participated in the study, with the majority aged 17 years (67.8%). A notable proportion of participants’ parents held a bachelor’s degree: 57.5% of mothers and 74.9% of fathers. Approximately 13.8% reported a family history of BC, and 73.6% of respondents identified social media as the primary source of BC information (Table 2).

**Table 2:** Demographical Characteristics of Participants *n* = 87

Question		*N* (%)*
Age (Years)	16	18 (20.7)
	17	59 (67.8)
	18	10 (11.5)
Mother’s Educational Level	Secondary	37 (42.5)
	Bachelor’s degree	50 (57.5)
Father’s Educational Level	Secondary	20 (22.9)
	Bachelor’s degree	65 (74.9)
	Higher Education	2 (2.2)
The Educational Level of the Recipient	Grade − 11	21 (24.1)
	Grade − 12	66 (75.9)
Monthly Family Income Average	High	17 (19.5)
	Moderate	70 (80.5)
Is there a family history of having breast cancer?	Yes	12 (13.8)
	No	64 (73.6)
	Not sure	11 (12.6)
Source of Information (Select all apply)	Family	28 (32.2)
	Social media	64 (73.6)
	Educational Curricula	44 (50.6)
	Media (TV, Radio)	21 (24.1)
	Friends and Relatives	22 (25.3)
	Books and Magazines	3 (3.4)
	Healthcare Professionals	13 (14.9)
Have you ever heard about breast cancer awareness?	Yes	43 (49.4)
	No	44 (50.6)

*Percentages are based on the total sample (*n* = 87)

#### School students’ perception and attitude

Pre-intervention, 80.5% of participants believed BC was curable, increasing to 98.9% post-workshop. The belief that early diagnosis increases the chance of recovery rose from 85.1% to 96.6%. Regarding attitudes toward seeking medical help, the proportion who would seek help immediately if they noticed abnormal breast changes rose from 56.3% to 74.7%. In comparison, those preferring to wait for symptoms to persist decreased slightly from 28.7% to 25.3%. Significantly, the proportion of participants reporting they would not seek help at all decreased from 13.8% to 0% following the intervention (Table 3).

**Table 3:** Changes in Perceptions and Attitudes of Participants Before and After Workshop

Question		Pre-workshop Agree *n* (%)	Post-workshop Agree *n* (%)	
Do you think breast cancer is a curable condition?		70 (80.5)		86 (98.9)
Do you think early diagnosis of breast cancer results in higher chances of recovery?		74 (85.1)		84 (96.6)
Do you think detecting breast cancer early results in lower mortality rates?		65 (74.7)		79 (90.8)
If you notice any abnormal changes in your breasts, will you seek medical help to know the cause?	Yes, if the symptoms persist for some time	25 (28.7)		22 (25.3)
	Yes, immediately	50 (57.4)		65 (74.7)
	No, I will not seek help.	12 (13.8)		0
If you do not seek immediate medical help, what is the reason? (Select all that apply) *	I do not have health insurance	4 (10.5)		2 (9.1)
	I feel embarrassed to address an issue in the breast area	15 (39.5)		9 (40.9)
	I am afraid of undergoing tests.	13 (34.2)		10 (45.5)
	I am afraid of receiving bad results.	17 (44.7)		11 (50.0)

Percentages are based on the total sample (*n* = 87)

*Percentages for these reasons are calculated based on the subgroup of participants who would not seek immediate medical help: pre-intervention *n*=38, post-intervention *n*=22

#### Knowledge gains across breast cancer signs, risk factors, and screening practices

Given the large number of items assessed, item-level McNemar tests were treated as exploratory, with primary emphasis on domain-level patterns (signs, risk factors, and screening practices). Detailed item-level results are provided in Supplementary Table S4 and are presented descriptively without adjustment for multiple comparisons. To convey the magnitude of knowledge gain, absolute percentage-point differences between pre- and post-workshop correct responses are reported for each item, illustrating the extent of knowledge gains across domains.

Baseline knowledge scores were compared across age groups and parental education levels using Kruskal–Wallis tests. No significant differences were found by age (*p* = 0.292), mother’s education (*p* = 0.421), or father’s education (*p* = 0.185), indicating similar pre-intervention knowledge across demographic groups.

Significant gains were observed in students’ recognition of less obvious breast cancer signs, such as nipple scaliness (34.5% to 75.9%, *p* < 0.001), abnormal discharge (27.6% to 85.1%, *p* < 0.001), and changes in breast shape (52.9% to 94.3%, *p* < 0.001). Awareness of unmodifiable risk factors, including early menarche, late menopause, and dense breast tissue, more than doubled (all *p* < 0.001). Knowledge of modifiable risks, such as obesity and physical inactivity, improved significantly (both *p* < 0.001), whereas awareness of hormonal therapy (*p* = 0.099) and smoking (*p* = 0.071) did not change significantly.

Awareness of breast self-awareness increased from 39.1% to 96.5% (*p* < 0.001); recognition that breast self-awareness aids early detection rose from 60.9% to 88.5% (*p* < 0.001). Knowledge of clinical breast exams improved from 48.3% to 69% (*p* = 0.003), and familiarity with mammogram imaging increased from 31.0% to 83.9% (*p* < 0.001). Students’ understanding that mammography should be conducted every two years improved from 19.5% to 43.7% (*p* < 0.001), and awareness that all women aged 40 years or older should undergo regular mammograms increased from 57.5% to 86.2% (*p* < 0.001).

Aggregated by domain, mean absolute percentage-point gains were + 31.0 for breast-cancer signs, + 23.6 for risk factors (reflecting overall improvement despite a slight decline for “hormonal therapy”), and + 34.8 for screening knowledge (Supplementary Table S4). Although these domains demonstrated substantial average improvements, the magnitude of item-level change varied, ranging from modest increases (+ 6.9) to large gains (+ 57.5) across Δ values.

**Fig. 2:**
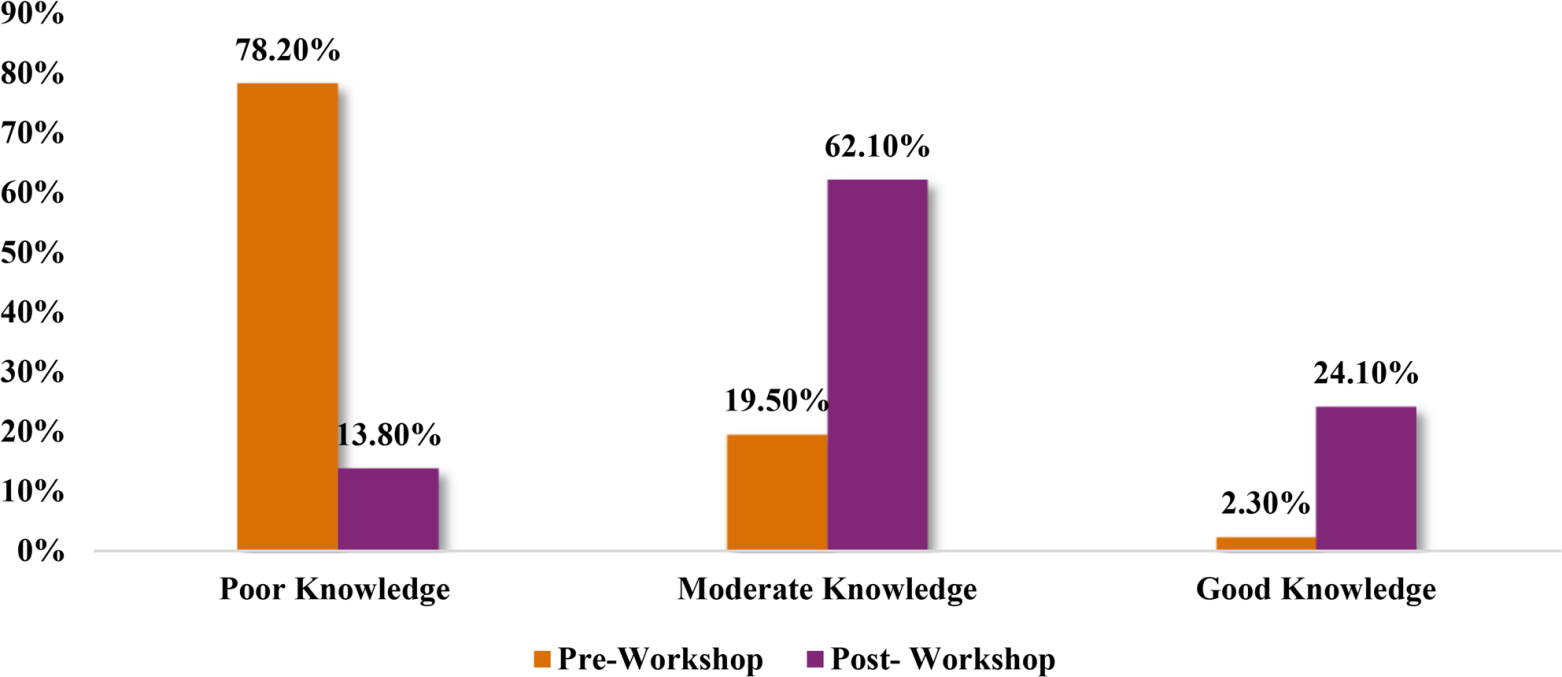
Comparison of Knowledge Mean Levels Pre and Post-Workshop

Normality tests (Kolmogorov–Smirnov and Shapiro–Wilk) on the difference scores indicated non-normality, justifying the use of the Wilcoxon signed-rank test. The analysis showed a significant increase in total knowledge scores (Z = 7.88, *p* < 0.001) with a large effect size (*r* = 0.84). The equivalent rank-biserial correlation (r₍rb₎ = 0.99) indicated that nearly all participants achieved higher post-test scores, reflecting a substantial educational impact. An effect size of *r* = 0.84, which is considered large by conventional benchmarks (0.1 = small, 0.3 = medium, 0.5 = large), reflects a substantial improvement in knowledge [57]. This large short-term effect size reflects substantial immediate post-intervention knowledge gains within this sample. However, given the single-group design and immediate assessment, these findings should be interpreted as preliminary indicators rather than definitive evidence of sustained educational impact. The mean total knowledge score increased from 41.7 ± 15.2 pre-workshop to 67.6 ± 15.7 post-workshop. Figure 2 illustrates the shift in participants’ knowledge categories (poor, moderate, and good) before and after the workshop.

### Integration of qualitative and quantitative findings

In this convergent mixed-methods design, triangulation of qualitative and quantitative data revealed a synergistic improvement in learning outcomes. Pharmacy students related enhanced communication skills, confidence, and professional identity, which aligned with significant knowledge gains and improved help-seeking attitudes among school participants. This meta-inference highlights the dual impact of student-led interventions in advancing both professional development and community health literacy.

## Discussion

This study explored the dual impact of a pharmacy student-led breast cancer awareness campaign conducted in secondary schools in Dubai, examining both its educational value for pharmacy students and its effectiveness in improving adolescents’ knowledge and health-seeking intentions. Four main themes were identified in the qualitative phase, corresponding to the four levels of the Kirkpatrick Model, and supported by Kolb’s experiential learning cycle. Quantitative findings demonstrated significant improvements in knowledge and attitudes among school participants, complemented by pharmacy students’ reflections of professional growth, advocacy skills, and self-directed learning.

### Experiential learning outcomes and curricular implications

This campaign provided a dynamic experiential learning opportunity that enabled pharmacy students to bridge theoretical coursework with meaningful community engagement. Grounded in Kolb’s experiential learning theory, the initiative guided students through the stages of planning, action, reflection, and adaptation, thereby deepening their understanding of BC awareness and public health competencies [58, 59]. The workshop functioned as a “concrete experience, while the qualitative reflection phase represented reflective observation and abstract conceptualisation, allowing students to evaluate their performance and identify areas for improvement.

These processes collectively facilitated active experimentation, illustrating how the campaign functioned as a complete experiential learning cycle. It is important to note that findings related to pharmacy students’ behavioural change and broader outcomes reflect self-reported experiences and intentions rather than observed or long-term behaviour change.

Students reported gaining essential transferable skills in communication, leadership, teamwork, and cultural competence, supporting their development as well-rounded, practice-ready graduates. The campaign also provided early exposure to authentic public engagement—a competency seldom emphasised in traditional classroom-based pharmacy curricula [60]. These outcomes mirror findings from other studies demonstrating that service-based learning and community outreach enhance empathy, civic responsibility, and self-directed professional growth [61].

Moreover, pharmacy students demonstrated clear cognitive and professional development. They reported increased confidence in their BC knowledge and improved ability to communicate complex information in age-appropriate and culturally sensitive ways. These competencies are essential for pharmacists in public-facing roles, particularly as the profession evolves toward preventive care and increased interprofessional collaboration [62].

Notably, many students experienced behavioural transformations that extended beyond the project. Several began practising breast self-awareness and discussing healthy habits with peers and family members, which represent self-perceived behavioural shifts rather than objectively measured behaviour change. This ripple effect reflects the profound influence of experiential learning in fostering advocacy, civic engagement, and a health-oriented identity. Such outcomes align conceptually with the higher tiers of the Kirkpatrick Model; however, in this study they reflect self-reported intentions and perceived impact rather than objectively measured behavioural or system-level change. The findings are similar toother student-led health initiatives, emphasising the possible long-term value of active participation in public health education [63].

The experiential outcomes align strongly with the CAPE 2013 Educational Outcomes framework [64]. Students demonstrated evidence-based communication and patient-centred education (Domain 2: Essentials for Practice and Care), advocacy for disease prevention and population health (Domain 3: Health and Wellness), and enhanced self-awareness, professionalism, and leadership (Domain 4: Personal and Professional Development). These achievements also correspond to FIP Competencies 1.3 (Patient Advocacy) and 2.4 (Interprofessional Collaboration), as well as to ACPE Standard 12, which underscores the importance of experiential education in developing practice-ready graduates [65, 66].

From a curricular perspective, embedding similar campaigns within pharmacy programs can transform outreach activities from isolated events into structured learning experiences. While this campaign was delivered as a co-curricular initiative, its educational and public health outcomes make a compelling case for formal curricular integration. Embedding the BC awareness campaign at multiple points along the experiential continuum strengthens curricular integration without increasing student workload. For example, a 15%-weighted project in Year 2 introductory experiential learning can serve as an initial exposure. At the same time, by Year 3, the campaign can evolve into a capstone where students design, implement, and evaluate expanded initiatives. By applying backward design principles, this campaign can be embedded across the curriculum to align with targeted competencies, such as public health advocacy. It also maps to Entrustable Professional Activities (e.g., EPA 3: educate patients and populations), supporting the progressive development of communication and health promotion skills [64, 67, 68].

In resource-constrained academic environments, student-led, school-based initiatives like this campaign demonstrate that meaningful, high-impact experiential learning can be achieved without extensive infrastructure [4, 69]. The success of this project illustrates that genuine educational reform is possible through thoughtful design, strong faculty mentorship, and community partnerships. This approach aligns with global pharmacy-education frameworks, including the FIP Global Competency Framework and ACPE standards, which highlight advocacy, health promotion, and communication as core pharmacist competencies. Both organisations emphasise sustained student participation in community-health initiatives integrated across longitudinal curricula rather than one-time outreach activities [64, 68].

Ultimately, this study suggestsculturally tailored experiential learning can serve as a potential model for integrating public health advocacy into pharmacy education. However, the present findings support the targeted integration of similar initiatives within pharmacy curricula, rather than universal implementation across all curricular stages, and further longitudinal and comparative research is needed to evaluate sustained learning outcomes and broader curricular impact.

### Adolescent outcomes

This campaign led to statistically tangible gains in schoolgirls’ knowledge of BC, particularly in recognising early symptoms and understanding risk factors. In line with previous research, participants demonstrated improved recognition of familiar and subtle BC signs, such as breast lumps, skin swelling, and abnormal nipple discharge, supporting the value of early educational interventions to promote symptom awareness and encourage timely medical consultation [43, 70–72].

Moreover, the campaign enhanced understanding of unmodifiable and modifiable risk factors. These findings align with earlier studies emphasising the critical role of health education in raising awareness of lifestyle-related and inherent risks [31]. However, awareness of certain factors—particularly hormone replacement therapy (HRT) and smoking—did not improve significantly. Although the campaign addressed common lifestyle risks such as diet and physical activity, these two topics were only briefly discussed. Recent meta-analyses indicate that current or recent use of HRT increases the risk of breast cancer, and almost half (48%) of women aged 20–70 in the UAE use HRT [73, 74]. The limited improvement in knowledge of smoking and HRT risk factors likely reflects this restricted content coverage. Future workshops should therefore incorporate explicit, culturally tailored content on these under-recognised yet clinically important risk factors.

Knowledge of screening practices improved markedly, with self-awareness nearly tripling and familiarity with clinical breast exams and mammography more than doubling, aligning with studies showing that awareness programs demystify preventive strategies and encourage screening uptake [75, 76]. The campaign also produced an 18.4% rise in participants’ willingness to seek medical advice for abnormal breast changes, addressing long-standing psychological and cultural barriers to early detection [77, 78]. This shift in intention, a key determinant of behaviour under the Theory of Planned Behaviour, suggests potential for increased screening over time, though sustained action will depend on social and systemic support [79].

In addition, caution is required when interpreting these results, as self-reported responses are susceptible to social desirability and recall bias, a recognised limitation in behaviour-change research [80]. Given the single-group, immediate post-intervention design, these significant effects should be interpreted cautiously, as substantial short-term gains are commonly observed in educational interventions without control groups.

### Cultural adaptations

Cultural adaptation played a crucial role in shaping students’ experiences and the campaign’s success. Delivering sessions in female-only settings and using culturally sensitive language enhanced students’ confidence in discussing breast health topics that are often considered taboo. This adaptation ensured respectful communication while enabling students to practise culturally competent health education, an essential skill for their future professional roles. Students described feeling empowered by their ability to navigate sensitive topics appropriately, highlighting the importance of cultural tailoring in maximising audience engagement and learning outcomes [81, 82].

### Challenges

Delivering the campaign required pharmacy students to navigate a combination of logistical, cultural, and pedagogical challenges common to school-based health promotion initiatives. Time constraints within academic timetables often limit the scope and depth of content delivery, a barrier widely reported in adolescent health education programmes [83]. Students also struggled to balance academic responsibilities with community engagement, reflecting the real-world tension between workload and outreach expectations. Furthermore, simplifying technical medical terminology for non-medical audiences without compromising accuracy required creativity and adaptability, reinforcing evidence that message tailoring is a critical competency for effective health communication [84, 85].

Despite these challenges, students demonstrated adaptability, teamwork, and problem-solving skills. Importantly, the campaign environment fostered interprofessional collaboration, enabling students to coordinate with teachers, faculty, and health professionals, modelling the cooperative practices expected in future pharmacy roles. These findings highlight the pedagogical value of confronting authentic barriers, as real-time problem-solving reinforces deeper learning and the development of transferable skills, such as cultural sensitivity and audience-centred education.

### Global relevance and educational impact

In the Gulf Cooperation Council (GCC) region, pharmacy student-led breast cancer (BC) initiatives remain limited, with most studies focusing primarily on knowledge assessments and social media engagement [21, 86–88]. By positioning students as both recipients and providers of health education, this initiative promoted reciprocal learning. Students consolidated their understanding through teaching, while adolescent participants benefited from relatable peer communication.

Beyond these individual-level gains, the campaign positioned pharmacy students as credible, accessible sources of health information. This emerging role can strengthen community trust in pharmacists as integral providers of preventive care and health advocacy, potentially facilitating greater public acceptance of pharmacists’ participation in cancer-screening and awareness activities. The intervention also served as a model for peer-delivered education in culturally diverse settings, demonstrating how school-based, adolescent-targeted initiatives can foster both individual empowerment and broader community health promotion. Evidence suggests that adolescents can act as conduits for health information within families, particularly in collectivist cultures where intergenerational dialogue strongly influences health decisions [89, 90].

Similar student-led initiatives across the Middle East and internationally reinforce the relevance and scalability of this approach. In Qatar and Oman [30, 91], Students have participated in interprofessional health campaigns and community clinics, developing communication and advocacy skills transferable to professional practice. In the United Kingdom, student pharmacists have designed and implemented real-life health-promotion campaigns within local communities, reporting strong perceived preparedness for professional roles in health advocacy and education [92]. In Malaysia, student-led public-health campaigns have resulted in significant gains in both content knowledge and communication confidence, demonstrating the educational value of authentic community engagement [93]. Similarly, in the United States, pharmacy students have directed school-based health-education programs that measurably improved children’s health knowledge while enhancing students’ confidence, professionalism, and advocacy skills [94].

Partnerships with Ministries of Health, professional bodies, and accreditation agencies, coupled with the use of existing educational resources, can further expand the reach of such initiatives while maintaining cost-effectiveness and curricular alignment. Collectively, the evidence highlights student-led experiential outreach as a sustainable, transferable model for developing practice-ready, socially accountable pharmacists equipped to advance community health worldwide.

## Strengths and limitations

The main strengths of this study lie in its mixed-methods design, which provided a comprehensive and multidimensional evaluation of both learning and public health outcomes. It integrated quantitative evidence of improved knowledge with qualitative insights into students’ experiential learning and professional development. The combination of pre- and post-intervention assessments with thematic analysis enabled both measurable outcomes and contextual understanding. Moreover, the use of the Kirkpatrick Model and Kolb’s experiential learning framework strengthened theoretical grounding and interpretation, while involving pharmacy students as campaign leaders demonstrated the feasibility of embedding community engagement within pharmacy education.

Despite these strengths, some limitations must be acknowledged. The study employed a single-group immediate pre–post design, which, while appropriate for exploratory educational evaluations, limits causal inference. Consequently, observed improvements should be interpreted as short-term knowledge gains associated with the intervention rather than definitive causal effects. Potential test–retest and social desirability biases may have influenced the results, particularly given that senior pharmacy students facilitated the sessions for younger peers. However, this peer-led approach was pedagogically intentional, aiming to promote relatable communication and mentorship within a real-world learning context.

The focus on private schools in Dubai limits the generalizability of findings to other educational and socioeconomic contexts. In addition, the modest sample size may have constrained statistical power, and reliance on self-reported surveys and focus group discussions may introduce response bias. A Hawthorne effect may also have influenced participants’ engagement and responses, as awareness of being observed can temporarily alter behaviour [95]. Furthermore, the follow-up period was limited, preventing assessment of the long-term sustainability of knowledge retention or behavioural change —a standard limitation in behavioural change studies [80].

The absence of member checking during qualitative analysis may have reduced participants’ opportunities to confirm interpretations, though triangulation across data sources helped strengthen credibility. Finally, the pharmacy students who participated may represent a self-selected group with greater motivation or advocacy inclination, limiting broader applicability, and they represented a subset of students in the health sciences.

## Conclusion

Within the bounds of its single-site design and modest sample, this mixed-methods study suggests that pharmacy student-led breast cancer awareness campaigns were associated with measurable improvements in adolescents’ knowledge and health-seeking intentions, as well as meaningful experiential learning outcomes among pharmacy students. Quantitative findings showed significant post-intervention gains in breast cancer knowledge and willingness to seek medical advice. At the same time, qualitative insights revealed growth across multiple levels of the Kirkpatrick Model, reflecting deeper reflection and emerging behavioural changes consistent with Kolb’s experiential learning framework.

The campaign served as both an educational and public health intervention, bridging theoretical learning with community engagement and equipping students with essential advocacy, communication, and cultural competence skills. Cultural tailoring, such as female-only delivery and adapted educational materials, was key to effective engagement, reinforcing the value of context-sensitive health education.

These findings support the integration of structured, student-led outreach within pharmacy curricula to meet international competency standards (FIP, ACPE, CAPE) and national accreditation goals for social accountability. At the policy level, student-led health campaigns may represent a promising, culturally adaptable approach that warrants further evaluation in broader, multi-site studies. Although the study assessed outcomes immediately following the intervention, participants’ reflections suggest potential for continued professional and behavioural growth. As this was an exploratory, short-term evaluation, future longitudinal or comparative studies are needed to assess the sustainability of these outcomes.

## Supplementary Information

Supplementary Material 1.

## Data Availability

Data will be available upon reasonable request from the corresponding author.
