# Supplementary material for: Evaluation of a student-led breast cancer awareness campaign as a co-curricular model in pharmacy education: a mixed-methods study in the UAE
Source: BMC Med Educ. 2026 May 11;26:1072. doi: 10.1186/s12909-026-09356-8 (PMC13335201; doi:10.1186/s12909-026-09356-8)
Supplement: Supplementary file 1 — Supplementary Material 1. [file 12909_2026_9356_MOESM1_ESM.docx]

**Table S1: Qualitative phase semi-structured interview guide**

- 1. Can you share your experiences and perspectives on being involved in planning, organizing, and conducting the breast cancer awareness campaign as a pharmacy student?
  2. What motivated you to participate in the awareness campaign?
  3. How do you feel the campaign impacted your knowledge and understanding of breast cancer?
  4. What were some challenges you encountered during the planning and execution phases of the campaign?
  5. Can you describe any memorable moments or interactions you had while engaging with secondary school females during the campaign?
  6. How do you perceive the role of pharmacy students in promoting breast cancer awareness among secondary school females?
  7. What are the most effective strategies for engaging and educating secondary school females about breast cancer?
  8. How do you think your involvement in the campaign has contributed to raising awareness about breast cancer in the community?
  9. What suggestions do you have for improving future breast cancer awareness campaigns led by pharmacy students?
  10. How do you envision the long-term impact of these awareness campaigns on the knowledge and attitudes of healthcare professionals and patients?

**Table S2: Quantitative phase pre- and post-workshop survey**

*Demographics*

1. Age: _________________________
2. Mother's educational level: School level/Bachelor's degree/Higher Education
3. Father's educational level: School level/Bachelor's degree/Higher Education
4. The Educational level of the recipient: _____________________
5. Monthly family income average: Low/Moderate/High
6. The primary sources of information and knowledge about breast cancer are: Family/Social media (Facebook, Twitter, etc.) /Educational curricula/Media (TV, radio)/Friends and relatives /Books and magazines /Healthcare professionals.
7. Is there a family history of having breast cancer? Yes/No/Not sure

*Knowledge about breast cancer, signs & symptoms, and associated risk factors*

1. Which of the following could be a sign of Breast cancer? (Choose all apply)

- Appearance of a lump in the breast or under the armpit.
- Increase in skin thickness or swelling in any breast area.
- Irritation or the presence of bumps on the breast skin.
- Redness or the appearance of scales on the nipple or its surrounding area.
- Pain or indentation in the nipple.
- Discharge from the breast other than milk.
- Changes in the shape or size of the breast.
- Pain in any area of the breast

1. Which of the following is considered a risk factor that may increase the risk of developing Breast Cancer (Choose all that apply)?

- Aging.
- Large breast size.
- Increased breast tissue density.
- Family history of the disease.
- Early onset of menstruation before the age of 11.
- Late onset of menopause after the age of 55.
- Not breastfeeding.
- Use of hormonal therapy.
- Sleep disturbances (such as insufficient sleep hours or irregular sleep patterns).
- Low-fiber diet (fiber-rich foods like whole grains, vegetables, and fruits).
- Carbohydrate-rich and high-glycemic-index foods (such as white bread and potatoes).
- Obesity (excess weight).
- Smoking.
- Secondhand smoke exposure (being in smoking areas).
- Stress.
- Lack of physical activity.

*Knowledge about the early detection and testing techniques of Breast cancer:*

1. Do you know what the concept of Breast self-awareness is? Yes/No/Not sure
2. Which of the following tests will help in the early detection of breast cancer? (choose all that apply)

- Breast awareness/Clinical diagnosis/testing (By physician)/Mammogram imaging

1. Do you think Breast awareness may enhance the early detection of Breast Cancer? Yes/No/Not sure
2. How often should a breast self-exam be conducted correctly?

Once per month/ Once per year/I am not sure

1. Are you familiar with breast imaging through a "Mammogram"?

Yes/No/Not sure

1. Do you think that mammogram imaging will help in the early detection of breast cancer?

Yes/No/Not sure

1. Mammography should be conducted every two years.

Yes/No/Not sure

1. Women who should regularly conduct mammography are. (choose all apply)

- Women with a family history of breast cancer.
- All women who have gone through menopause (post-menopausal).
- All women who are 40 years of age or older

1. If you notice abnormal breast changes, will you seek medical help to know the cause?

- Yes, immediately.
- Yes, if the symptoms persist for some time.
- No, I will not seek help

1. If NOT, please mention why

- I don't have enough time.
- I do not have health insurance.
- I feel embarrassed to address an issue in the breast area.
- I am afraid of undergoing tests.
- I am afraid of receiving bad results.
- Other reasons

*Participants attitude*

1. Would you be interested in participating in seminars or conferences focused on breast cancer awareness and the significance of early detection in the future? Yes/No/Not sure
2. Do you think breast cancer is a curable condition? Yes/No/Not sure
3. Does early diagnosis of breast cancer result in higher chances of recovery? Yes/No/Not sure
4. Does detecting breast cancer early result in lower mortality rates? Yes/No/Not sure,
5. do you feel more motivated to read and research the topic after attending the awareness lecture? Yes/No/Not sure
6. After attending the breast cancer awareness lecture, do you plan to inform your family and relatives, especially women over 40, about the importance of regular and early screening through mammograms? Yes/No/Not sure

**Table S3: COREQ Checklist**

| Domain | Topic | Item No. | Description | Reported |
| --- | --- | --- | --- | --- |
| Domain 1: Research Team and Reflexivity | Personal Characteristics | 1 | Which author/s conducted the interview or focus group? | Azhar T Rahma, Iffat Elbarazi |
|  |  | 2 | What were the researcher's credentials? | PhD |
|  |  | 3 | What was their occupation at the time of the study? | Instructor |
|  |  | 4 | Was the researcher male or female? | Female |
|  |  | 5 | What experience or training did the researcher have? | Comprehensive training in qualitative methods, including thematic analysis and interview experience. |
|  | Relationship with Participants | 6 | Was a relationship established prior to study commencement? | No |
|  |  | 7 | What did the participants know about the researcher? | Academic background, study objectives, impact on healthcare and insurance policies. |
|  |  | 8 | What characteristics were reported about the interviewer/facilitator? | Background in public health, qualitative research experience. |
| Domain 2: Study Design | Theoretical Framework | 9 | Methodological orientation stated to underpin the study? | Thematic analysis, Kirkpatrick model |
|  | Participant Selection | 10 | How were participants selected? | Purposive, convenience |
|  |  | 11 | How were participants approached? | Online meetings |
|  |  | 12 | How many participants were in the study? | 16 |
|  |  | 13 | How many people refused to participate or dropped out? Reasons? | None |
|  | Setting | 14 | Where was the data collected? | Virtually via Microsoft Teams |
|  |  | 15 | Was anyone else present besides the participants and researchers? | No |
|  |  | 16 | Important characteristics of the sample? | The pharmacy student who conducted the breast cancer awareness campaign |
|  | Data Collection | 17 | Were questions/prompts provided? | Yes, developed from literature and expert feedback. |
|  |  | 18 | Were repeat interviews carried out? | No |
|  |  | 19 | Was audio/visual recording used? | Yes |
|  |  | 20 | Were field notes made? | No |
|  |  | 21 | Duration of interviews/focus groups? | 60-90 minutes |
|  |  | 22 | Was data saturation discussed? | Yes |
|  |  | 23 | Were transcripts returned to participants for comment and/or correction? | No |
| Domain 3: Analysis and Findings | Data Analysis | 24 | Number of data coders? | 2 |
|  |  | 25 | Was a coding tree description provided? | Yes |
|  |  | 26 | Were themes identified in advance or derived from the data? | Derived from data |
|  |  | 27 | What software was used to manage the data? | NVivo |
|  |  | 28 | Did participants provide feedback on the findings? | No |
|  | Reporting | 29 | Were participant quotations presented to illustrate findings? | Yes |
|  |  | 30 | Was there consistency between data and findings? | Yes |
|  |  | 31 | Were major themes clearly presented? | Yes |
|  |  | 32 | Were minor themes described or discussed? | Yes |

Table S4: Participant Knowledge of Breast Cancer Before and After Workshop

| Variable | | | | Pre-workshop  correct answer  n (%) | Post-workshop  correct answer  n (%) | | | McNemar  P value | | | Δ (percentage-points) |
| --- | --- | --- | --- | --- | --- | --- | --- | --- | --- | --- | --- |
| Breast Cancer Signs Knowledge | | | |  |  | | |  | | |  |
| Appearance of a lump in the breast or under the armpit | | | | 72 (82.8) | 82 (94.3) | | | 0.013 | | | +11.5 |
| Increase in skin thickness or swelling in any breast area | | | | 45 (51.7) | 63 (72.4) | | | 0.004 | | | +20.7 |
| Irritation or the presence of bumps on the breast skin | | | | 38 (43.7) | 65 (74.7) | | | <0.001 | | | +31.0 |
| Redness or the appearance of scales on the nipple or its surrounding area | | | | 30 (34.5) | 66 (75.9) | | | <0.001 | | | +41.4 |
| Pain or indentation in the nipple | | | | 37 (42.5) | 64 (73.6) | | | <0.001 | | | +31.1 |
| Discharge from the breast other than milk | | | | 24 (27.6) | 74 (85.1) | | | <0.001 | | | +57.5 |
| Changes in the shape or size of the breast | | | | 46 (52.9) | 82 (94.3) | | | <0.001 | | | +41.4 |
| Pain in any area of the breast | | | | 63 (72.4) | 75 (86.2) | | | 0.023 | | | +13.8 |
| Breast Cancer Risk Factors Knowledge | | | |  |  | | |  | | |  |
| Unmodifiable Risk Factors | | Ageing | | 45 (51.7) | | | 68 (78.2) | | | <0.001 | +26.5 |
|  |  | Increased breast tissue density | | 29 (33.3) | | | 64 (73.6) | | | <0.001 | +40.3 |
|  |  | Family history of the disease | | 43 (49.4) | | | 50 (57.5) | | | 0.024 | +8.1 |
|  |  | Early onset of menstruation before the age of 11 | | 11 (12.6) | | | 44 (50.6) | | | <0.001 | +38.0 |
|  |  | Late onset of menopause after the age of 55 | | 17 (19.5) | | | 62 (71.3) | | | <0.001 | +51.8 |
| Modifiable Risk Factors (Lifestyle) | | Not breastfeeding | | 28 (32.2) | | | 46 (52.9) | | | <0.001 | +20.7 |
|  |  | Use of hormonal therapy | | 35 (40.2) | | | 30 (34.5) | | | 0.099 | –5.7 |
|  |  | Lack of physical activity | | 28 (32.2) | | | 54 (62.1) | | | <0.001 | +29.9 |
|  |  | Obesity (excess weight) | | 32 (36.8) | | | 54 (62.1) | | | <0.001 | +25.3 |
|  |  | Smoking | | 31 (35.6) | | | 43 (49.4) | | | 0.071 | +13.8 |
| Potential Risk Factors | | Low-fibre diet (foods rich in fiber like whole grains, vegetables, and fruits) | | 21 (24.1) | | | 38 (43.7) | | | <0.001 | +19.6 |
|  | | Sleep disturbances (such as insufficient sleep hours or irregular sleep patterns) | | 17 (19.5) | | | 40 (46.0) | | | 0.015 | +26.5 |
|  | | Carbohydrate-rich and high-glycemic-index foods (such as white bread and potatoes) | | 20 (23.0) | | | 58 (66.7) | | | <0.001 | +43.7 |
|  | | Second-hand smoke exposure (being in smoking areas) | | 25 (28.7) | | | 31 (35.6) | | | 0.307 | +6.9 |
|  | | Stress | | 16 (18.4) | | | 31 (35.6) | | | 0.006 | +17.2 |
| Misconception | | Large breast size | | 57 (65.5) | | | 70 (80.5) | | | 0.019 | +15.0 |
| Breast Cancer Screening Knowledge | | | | | | | | | | |  |
| Do you know what the concept of breast self-awareness is? | | | 34 (39.1) | | | 84 (96.5) | | | <0.001 | | +57.4 |
| Which one of the following will help in the early detection of breast cancer? | Breast self-awareness | | 53 (60.9) | | | 77 (88.5) | | | <0.001 | | +27.6 |
|  | Clinical examination | | 42 (48.3) | | | 60 (69.0) | | | 0.003 | | +20.7 |
|  | Mammogram imaging | | 32 (36.8) | | | 59 (67.8) | | | <0.001 | | +31.0 |
| Are you familiar with the concept of breast imaging by “Mammogram”? | | | 27 (31.0) | | | 73 (83.9) | | | <0.001 | | +52.9 |
| Do you think that mammogram imaging will help in the early detection of breast cancer? | | | 50 (57.5) | | | 81 (93.1) | | | <0.001 | | +35.6 |
| Mammography should be conducted every two years. | | | 17 (19.5) | | | 38 (43.7) | | | <0.001 | | +24.2 |
| Women who should regularly conduct mammography are: | All women who are 40 years of age or older | | 50 (57.5) | | | 75 (86.2) | | | <0.001 | | +28.7 |

**Percentages are based on the total sample (n = 87).*
